# Development and Characteristics of a Dual-Layered Vascular Phantom

**DOI:** 10.1007/s13239-025-00810-0

**Published:** 2025-11-10

**Authors:** Kaitlyn M. Elmer, Cassidy Caffin, Breeanna Scott, Sam E. Stephens, Morten O. Jensen

**Affiliations:** 1 Department of Biomedical Engineering, University of Arkansas, Fayetteville, AR USA; 2 Department of Surgery, University of Arkansas for Medical Sciences, Little Rock, AR USA

**Keywords:** Phantoms, Gels, Multi-layer phantoms, Tissue mimics, 3D printing, Resin printing, Vat photopolymerization

## Abstract

**Purpose:**

Cardiovascular phantoms are used in biomedical research and development applications, allowing for complex geometries to be studied in a controlled environment. The various layers of human tissue have been difficult to mimic in these phantoms. In this study, a novel dual-layer cardiovascular phantom is created.

**Methods:**

The interior lumen is 3D printed using an elastic vat-photopolymerization resin and cast within an industry standard tissue-mimicking ballistics gel. Strips of the 3D-printed resin were prepared and tested to determine Young’s modulus, Ultimate tensile strength, and elongation at break.

**Results:**

The final phantoms were reproducible, semi-transparent, and suitable for microCT scanning. Additionally, the 3D-printed elastic materials had: Young’s Modulus of 12 +/- 3.2 MPa, UTS of 1.27 +/- 0.44 MPa, and elongation at break of 29 +/- 9%. These results are within the physiological ranges of human tissues. There was a moderate correlation between the thickness of the sample and stiffness, which may be important depending on the application of the models.

**Conclusion:**

The methods for producing a dual-layered phantom are reproducible and appropriate for a variety of biomedical applications.

## Introduction

Benchtop phantoms that mimic vascular structures are widely used in biomedical research. Uses for tissue mimics include pre-clinical testing of medical devices, developing new techniques for device delivery, hemodynamic studies, and training of medical personnel [1–7]. Tissue-mimicking phantoms vary depending on the end-use of the phantom. Physiological structures such as coronary, carotid, and cerebral arteries, cerebral aneurysms, the aortic arch, and heart ventricles have all been mimicked in phantoms from simple tube-shapes to patient-specific models meshed from clinical data [2, 4–6, 8–14]. Models of increasing similarity to in-vivo geometry tend to be preferred [4]. Other desirable characteristics are similar mechanical properties to in-vivo tissue, and imaging capability. For example, particle image velocimetry (PIV) imaging requires optical clarity and refractive index matching to the working fluid, ultrasound imaging uses the speed of sound through the material, and clear microCT scanning depends on density variations [3, 12]. Imaging modality also depends on the end use of the phantom; PIV is often used in flow studies while microCT may be used for checking device placement [3, 12–14].

Many methods exist for producing single-layer phantoms. One style of single-layer phantom is a block phantom, or “wall-less” phantom, constructed as a block of tissue-mimicking material with voids within to mimic the desired vessel [1, 4, 5, 12, 15, 16]. The simplest of these can be channels cut into acrylic, used for benchtop testing of cardiovascular stents [15]. However, compliant, tissue-mimicking materials are preferred. Block phantoms are typically cast around a removable core [1, 4, 5, 12, 16]. Block phantoms are stable due to the large surrounding tissue block, and do not require external support to hold the shape of the vessel. However, block phantoms use a single layer and lack a vessel wall, which does not fully mimic the physiology of the cardiovascular system. Walled phantoms, another form of single-layer phantom, use a thin-walled, vessel-mimicking layer, which may be constructed with multiple methods. Many are 3D printed or cast with compliant materials [4, 9, 17–22]. Others have been constructed using a spin-dip coating method [23]. These phantoms also are only one layer, and typically require external supports to be held in the desired position.

Molded phantoms use a core and outer mold. The simplest cores use metal rods which can slide from the finished phantom, sometimes in multiple parts, machined to fit together [8, 14]. More complex cores geometries may be 3D printed in a solvent-soluble material, such as ABS (acetone-soluble) or polyvinyl alcohol (PVA) [8, 12, 13, 16, 23]. Other cores have been cast in materials like plaster, Aquapour, and even chocolate [1, 24]. Low-density polylactic acid (PLA) prints have been used, and removed by carefully destroying the PLA and removing from the mold [2]. The chosen core material must be compatible with the phantom material. For example, oil-based gels are not compatible with ABS, as they are cast by melting the gel at temperatures that soften ABS. Additionally, cores tend to require additional surface work to smooth imperfections from print layer lines [1, 5, 8, 12, 23].

The outer mold for a molded phantom can be a simple block for wall-less phantoms, or more complex for walled phantoms. Molds can be 3D-printed or machined from materials like aluminum [14]. Molded phantoms with curvature in multiple planes may be difficult to achieve; for example, a tortuous walled vessel would be difficult to produce without undercuts in the mold. Phantoms have also been constructed without molds, using painting or dipping methods over a soluble core [13, 23]. Phantoms that are produced using dip or spin-dip methods are difficult to control wall thickness, and complex geometries often require manual touch-ups in the silicone [13, 23]. Spin-dipping can aid with wall-thickness control, though it often does not completely solve the issue and can add additional technical complexity [23].

Compliant materials used for tissue include gelatin and agar solutions, artificial oil-based gelatins, polydimethylsiloxane (PDMS) or other silicones, and polyvinyl acetate cryogels [1–3, 5–8, 10–14, 23]. Gelatin, agar and PDMS can be mixed at different ratios to tune mechanical properties [2, 5, 12, 24, 25]. PVA-c gels may be stiffened using multiple freeze-thaw cycles [2, 3, 10]. Oil-based gelatins and silicones are available in different stiffnesses [1, 8]. Gelatin, agar, and PVA-c are not shelf-stable, eventually dehydrating. PVA-c can also require multiple freeze-thaw cycles at 24 h each to reach the desired stiffness. Selected mechanical properties for many of these materials are available in Appendix I. Trapped air bubbles, which in addition to being unsightly, often affect bulk phantom mechanical properties, can make casting processes difficult; vacuum chambers are sometimes used to counteract this [8, 11, 14].

Additive manufacturing has also been used for phantom production [9, 17–22, 26, 27]. Several compliant materials are available with photopolymerizable 3D printers. For example, vat photopolymerization printed flexible resins have been demonstrated to develop a workflow for segmenting patient-specific aortic aneurysms and printing [27]. Another group has developed coatings to increase transparency for flexible resins [26]. Other materials are available, including inkjet photopolymerization with Stratasys’ line of compliant materials which have been used to mimic the aorta and other elements of the coronary tree [18–20, 22]. Compliant 3D-printed materials are available in different stiffnesses, summarized in Appendix I.

Multi-layer phantoms are highly sought after, as vessels in-vivo have multiple layers and are imbedded in tissue, with each component composed of different mechanical properties [28–34]. Successive layers of silicone have been injection-molded around increasingly larger aluminum molds [14]. Another approach has been to make walled carotid phantoms, casting a PVA-c vessel in an agar-gelatin mixture [2]. However, these approaches use materials that are not shelf-stable, and materials that require multiple freeze-thaw cycles of 24 h each. Molded multi-layer phantoms are also subject to the same molding difficulties for complex shapes, including undercuts and producing complex, removable cores [14].

There is a need for a new method capable of creating both shelf-stable and custom-geometry walled vascular phantoms that are simple to prepare, accurately mimic tissue properties, and are convenient for biomedical and clinical research applications. To our knowledge, no true multi-layer phantoms have yet been achieved with shelf-stable, tissue-mimicking materials. Our overall objective in this work is to develop and describe a process for embedding a vessel wall model within a tissue-mimicking material, creating phantoms of complex geometry with two distinct layers. A previously-developed method for casting voids in artificial gelatin is modified to incorporate an inner wall created by additive manufacturing [8]. The oil-based gelatin’s mechanical properties have been described previously and are within physiological ranges [8]. Casting multiple, distinguished layers of oil-based gelatins is quite difficult; though the gels are available in different stiffnesses that can be melted and cast into the desired shape, they all melt at approximately the same temperature (Humimic Medical, Greenville SC). Thus, an inner lumen material that can be simply imbedded within a gel layer is desired. Newer materials for vat photopolymerization (VPP) printing are compliant and straightforward to work with, showing promise for use in vessel wall models. Formlabs “Elastic” and “Flexible” resins (Formlabs, Somerville MA) have been used to create compliant single-layer vessel wall phantoms [17, 21, 26]. Thus, this work seeks to imbed a 3D-printed compliant lumen within a tissue-mimicking gel. The “Flexible” resin’s ultimate tensile strength (UTS) was reported by the manufacturer to be much higher than literature values for human arterial wall UTS, so for simplicity only the elastic resin is used in this study. A previous version of the VPP resin (“Elastic V1”, Formlabs, Somerville MA) has mechanically characterized, but the recently released “Elastic V2” has not yet been reported to our knowledge [21, 26]. So, a secondary objective in this work is to conduct tensile testing with the “Elastic V2” resin.

Finally, preliminary work on the applicability of the phantom for biomedical testbeds is demonstrated. MicroCT scanning is highly relevant for medical device implantation, showing the location of implanted medical devices within vascular models. Our first imaging work is with microCT scanning of a prototype coronary stent and subsequent segmenting.

## Methods

### Phantom Fabrication

**Fig. 1:**
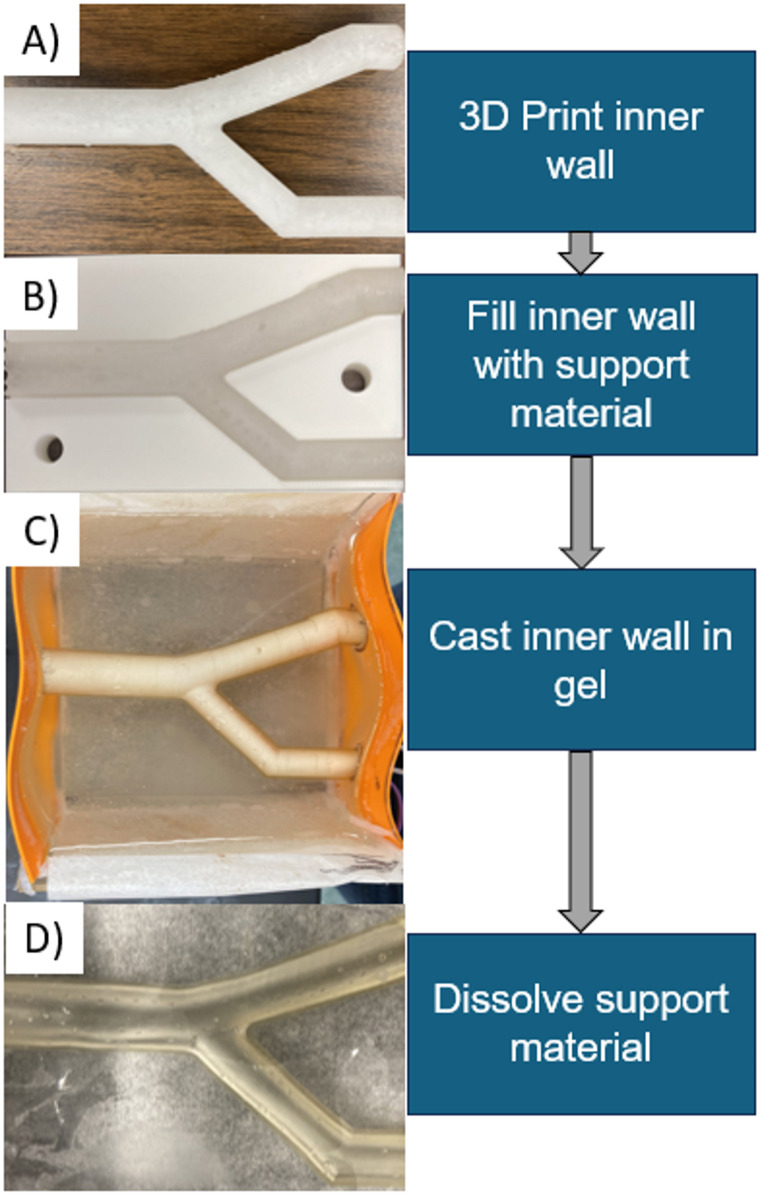
Two-layer vascular model construction. (**A**) The compliant artery wall was printed using an elastic resin, and (**B**) filled with a castable ceramic material for support. Once the support material was hardened, (**C**) the artery was imbedded in the gel, and (**D**) the support material was dissolved

The phantom fabrication process is composed of three sub-processes: (1) Creation of the walled vessel; (2) Imbedding of walled vessel in artificial gelatin (3) Dissolution of Insert Supports. The process is summarized in Fig. 1.

First, the internal wall is designed in 3D modelling software (Solidworks, Dassualt Systems, Vélizy-Villacoublay, France) and printed with Elastic V2 resin (Formlabs, Somerville MA) using vat photopolymerization additive manufacturing. According to the manufacturer, the resins are chemically stable up to 250° C, which is above the temperature used to melt and de-bubble gels (225° C). The artery walls for the elastic resin were at least 1 mm in thickness, per the manufacturer’s recommendations. The artery wall was created by designing a solid core, then outwardly shelling to create the hollow artery wall.

Once printed and cured, support material is snipped off and the vessel wall is lightly sanded to remove bumps from support material. To embed the vessel in the gel, the vessel wall is stabilized with a water-soluble casting material (Aquapour, Advanced Ceramics Manufacturing, Tuscon AZ). Aquapour is prepared according to manufacturer’s instructions, using a 0.8 ratio of powder to water. The internal support is poured into the vessel or injected with a luered syringe and left to harden for at least two hours.

The vessel is placed in a casting mold, either before or after Aquapour filling. For larger diameter vessels (> 10 mm), the vessels are first stabilized with Aquapour to avoid sagging. For smaller vessels with more complex shapes, the vessels were filled after placing in the mold to avoid manipulating the stiffened vessel. Vessels were extended at their ends to pass through holes in the mold sides, securing them within the mold. The intersection between the vessel and the gel mold is sealed with silicone caulk. Tissue-mimicking gel (Humimic Medical, Greenville, SC) was heated and poured into molds; then the entire casting mold with gel was heated in an oven to de-bubble, usually 2–3 h. Additional details on casting molds used are available in Appendix I. Bubbles on the surface of the gel were easily cleared using a heat gun, and the gel cooled at room temperature. Once cooled, the final steps are dissolving the inner core with warm water from a low-pressure hose and removing the assembled phantom from the casting mold. Figure 1 shows the entire process with an idealized coronary bifurcation phantom. In this study, three idealized bifurcation vessels and one patient-specific coronary artery were constructed using the described method.

### Phantom Geometries

**Fig. 2:**
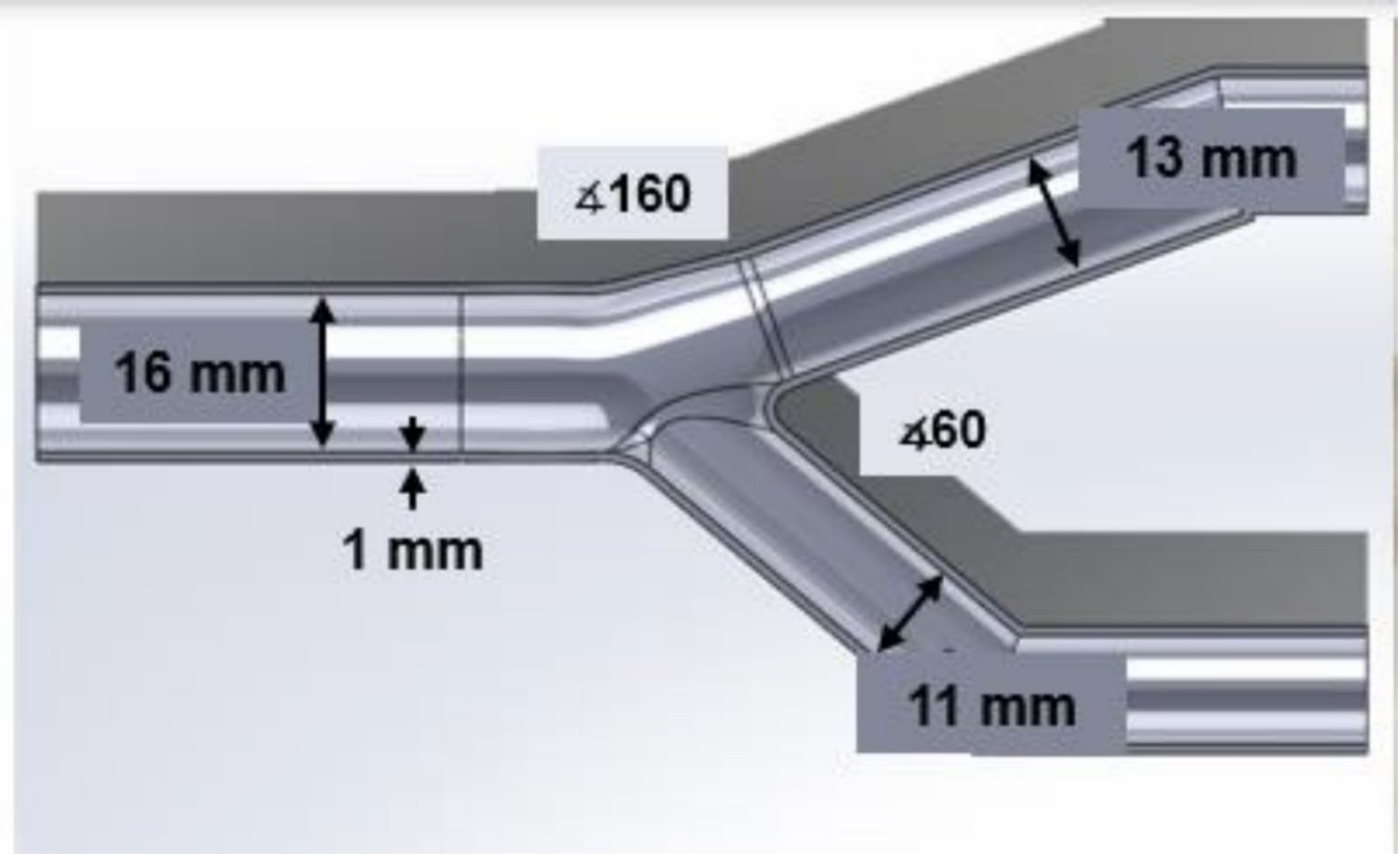
Idealized bifurcation phantom dimensions, 16 mm ID of the proximal vessel, 13 mm ID of the distal vessel, and 11 mm ID of the side branch. 60 degrees between the distal vessel and side branch, 160 degrees between the proximal and distal vessels

Two geometries were created with this method. First, three scaled phantoms for an idealized coronary bifurcation were produced. The phantom design is shown in Fig. 2.

These angles were chosen based on averages of coronary bifurcation shape, recommended in consensus documents [4]. The vessel segment diameters are scaled 5x from average sizes of a coronary bifurcation; maintaining an anatomically correct Finet’s ratio of 0.67. Scaling was done to fit available stent prototypes for microCT imaging, which were constructed in-house and described previously [35].

**Fig. 3:**
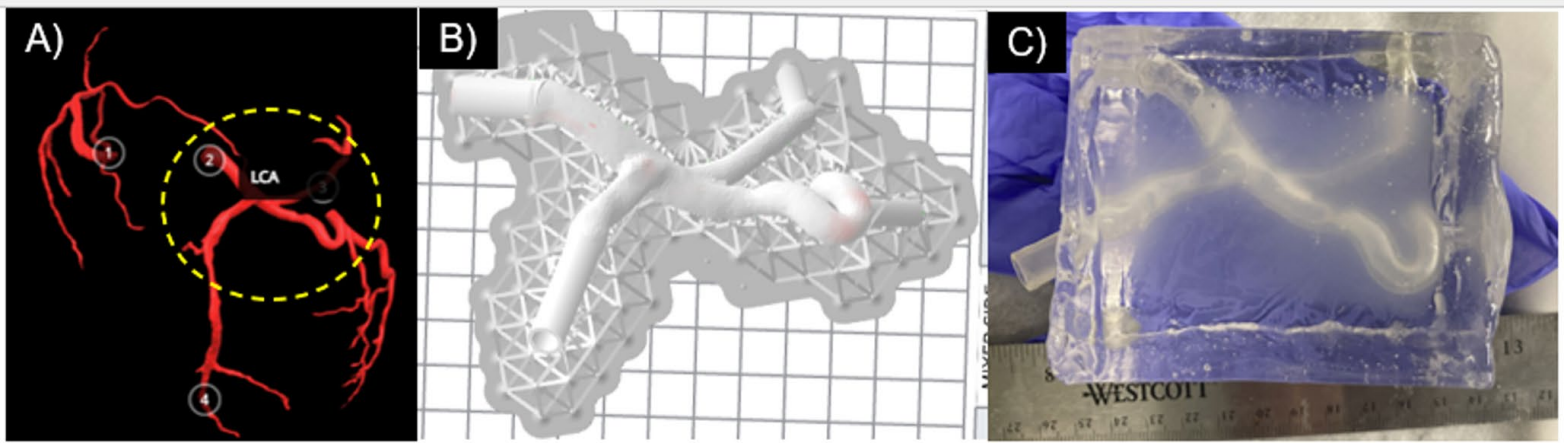
Patient-specific artery model encapsulated in tissue-mimicking gel. (**A**) The entire coronary tree directly from the Atlas, LCA region of interest circled. (**B**) LCA prepared for printing in Formlab’s printing software, with inlet/outlet geometry lengthened. (**C**) Completed phantom

The second tested geometry was a patient-specific model of the left coronary artery (LCA). The patient-specific model used the same method as described previously, except for additional steps in the print file preparation. The patient-specific model was sourced from the Atlas of Human Cardiac Anatomy, used under a Creative Commons license [36]. Models of the coronary tree are segmented from perfusion-fixed hearts and are available for download as .stl files [36]. Heart 0183 (Female, Age 49, 78.6 kg body mass, 157.5 cm height), shown in Fig. 3A, was downloaded and prepared in Meshmixer (Autodesk, San Francisco CA). The model was trimmed to include the left coronary artery (LCA), the base of left circumflex (LCX), a left marginal artery (LMA), and the base of the left anterior descending (LAD). This was chosen to test the above-described method with a more complex artery shape, which includes three branches, and a tortuous left marginal artery. The artery was shelled outward in Meshmixer to have a wall thickness of 1 mm. The ID of the arteries were: LCA 6.2 mm; LAD 5.1 mm; LMA 5.5 mm; LCX 4 mm. The vessel ends were then extruded as tubes in Solidworks to be compatible with our casting molds and standard luer-lock attachments.

### MicroCT Scanning

The three idealized coronary phantoms were tested with prototype stents and then microCT scanned to assess compatibility with microCT scanning. First, guidewires were visually fed into the bifurcation, and a prototype stent and balloon-catheter were delivered to the bifurcation models. These prototypes are described previously [37]. The phantoms with the embedded stents were microCT scanned, and the image stacks oriented using Matlab (Mathworks, Natick, MA) and ImageJ [38] to the cross section of the bifurcation. Scanned phantoms were segmented and examined to determine if the contrast between the stent, arterial layer, and gel layer was sufficient for future testing. The circular cross-section of the distal vessel was used as a fiducial marker, with the amount of ellipticity in the cross section used to calculate an angle of rotation. Once rotated in ImageJ, a second set of vessel cross-sections from different locations confirmed that the cross sections were circular and that the center of the cross-sections was in a consistent location. Additionally, the scanned phantom walls were observed to determine smoothness of the inner and outer walls of the printed lumen. Finally, previous tests with the single gel layers showed that the prototype stents ripped the vessel wall, so the resistance to mechanical wear-and-tear of the two-layer models was also observed.

### Mechanical Testing of Materials

Rectangular specimens of length = 82 mm, width = 25 mm, and a varied thickness were prepared in the Elastic V2 resin for mechanical testing. Varying thicknesses were chosen to correspond to literature values of vessel thicknesses [39]. Small, medium, and large sizes of arteries and veins were chosen, according to values shown in Table 1. Six specimens for each size were created (*n* = 6).

**Table 1:** Literature blood vessel thickness values and thickness of elastic test specimens [39]

Blood Vessel Type	Literature Values (mm)	Specimen Thicknesses (mm)
Large Artery (LA)	1.5	1.5
Medium Artery (MA)	1.0	1.0
Small Artery (SA)	0.125-0.8	0.46
Large Vein (LV)	1.2	1.2
Medium Vein (MV)	0.8	0.8
Small Vein (SV)	0.04–0.5	0.27

Specimens were designed in 3D modelling software and arranged vertically for printing with layer lines perpendicular to the strain axis. Elastic V2 resin was used at it was the softest available material for use with Form 3 printers, though “Flexible” resin was also initially explored. Samples were printed on a Form 3 printer and post-processed according to the manufacturer’s recommended settings for washing and curing parts for half of all samples, which included submerging the samples in a water bath during curing. The other half of the samples were cured in air rather than with the recommended water submersion to test the resulting effect on mechanical properties. In other words, for each thickness, half of the samples were cured within a transparent 1 L beaker of water and half were cured without water. Curing without water left the samples with a tacky finish, which may encourage better adhesion between the gel and vessel wall.

#### Uniaxial Tensile Testing

Uniaxial tensile testing was conducted using an Instron machine at a strain rate of 50 mm/min (Instron, Norwood, MA). Each sample was measured prior to testing to determine inputs to length, width, and height. The samples were secured within the clamps and centered. Stress and elongation were both zeroed before each test. Each sample was tested until failure, or for three trials if the sample slipped. If a sample slipped from the clamps, the specimen was re-measured and re-tested. No preconditioning was conducted. Results were exported to an Excel file for analysis.

#### Analysis of Tensile Tests

The tensile testing output presents force and elongation in 0.05 s intervals. Engineering stress and strain were calculated for each test run according to Eqs. 1 and 2:

1 $$\:\sigma\:=\frac{F}{{A}_{o}}$$

2 $$\:\epsilon\:=\frac{{L-L}_{0}}{{L}_{0}}=\frac{\varDelta\:L}{{L}_{0}}$$

Where:

$$\sigma\:$$ = stress.

F = force.

${A}_{o}$ = initial cross-sectional area.

$$\epsilon\:$$ = strain.

L = length at time interval.

$${L}_{0}$$ = initial length.

The stress-strain curves were then plotted. The linear portion of the stress-strain curves were identified manually, and a straight line was fit to the linear regions using a regression analysis. R-squared values were calculated to ensure linearity, with $\:{r}^{2}$>0.9 used as acceptance criteria. The elastic modulus (E) is defined as the slope of the stress-strain curve. The Young’s moduli from each cure process and thickness were compared using unpaired, two-tailed T-Tests with a *p*-value of 0.05.

## Results

### Phantoms

Three phantoms of an idealized coronary bifurcation and one patient-specific vessel were successfully produced using the described methods. De-bubbling of the idealized gel phantoms was observed to be a quicker process than with previous methodology [8]. We hypothesize that the idealized phantom lumen worked better than previous methods with a single PVA core because the dissolvable PVA emitted gas during gel heating. Next, sanding the outside of the vessel removes the print layer lines, which helps air bubbles not to become trapped. Meanwhile, the castable mandrel material (Aquapour) is permeable, allowing air inside the vessel to escape through the ends of the insert rather than expanding within the phantom and causing deformation of the interior layer. The interior arterial layer did not adhere to the surrounding gel, though there was high friction between the gel and lumen, and the lumen could not easily be pulled from the gel without ripping.

**Fig. 4:**
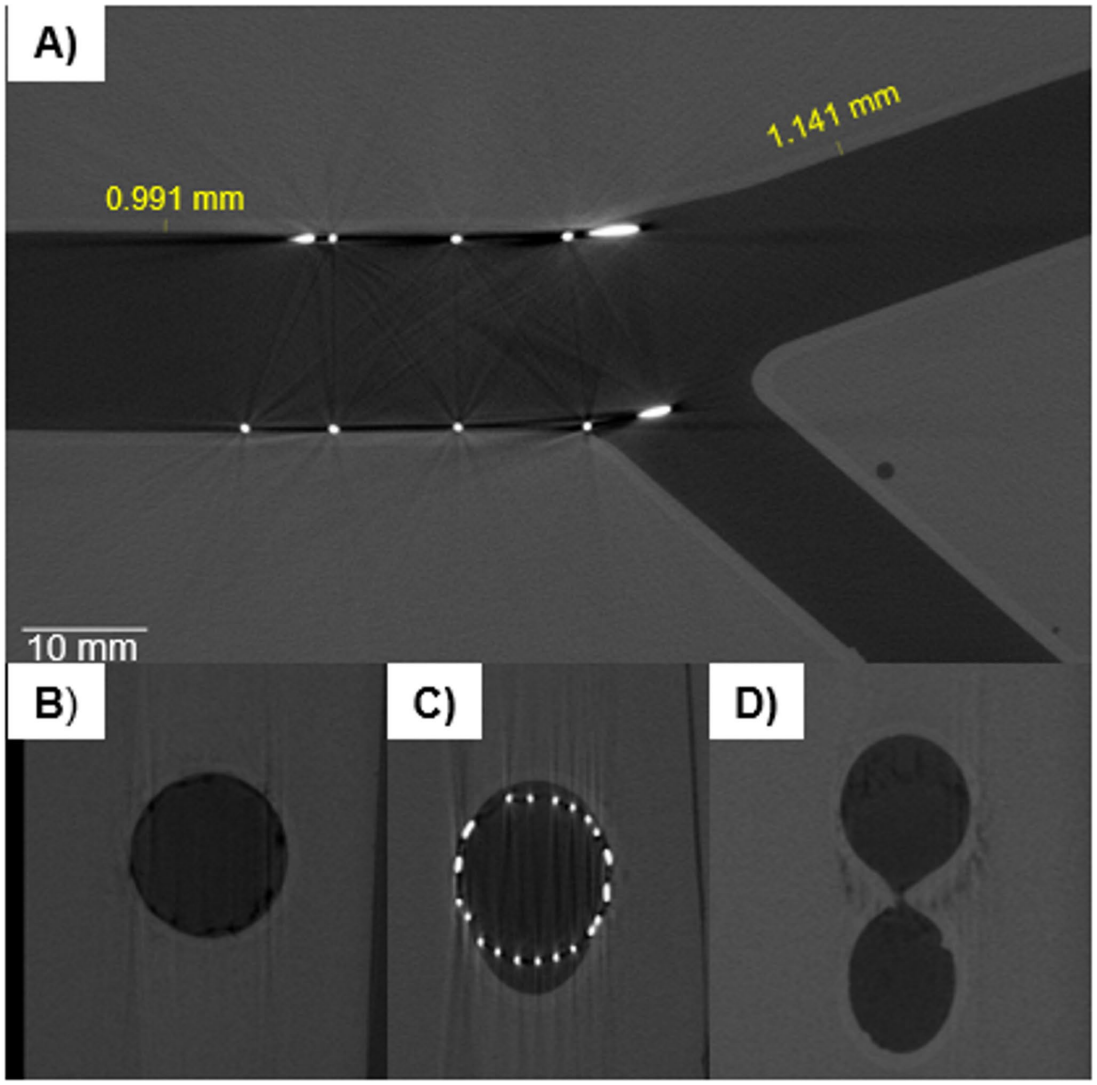
MicroCT scan of stent placed in dual-layer arterial phantom. The internal vessel (light gray), phantom (medium gray), void (darkest gray), and stent (bright white) were all visible in the scan. The scans also showed smooth lumens where the support material was trimmed and sanded. **A**) shows a transverse cross-section of the bifurcation. **B**-**D** show vessel cross sections in the proximal vessel (**B**), near the side-branch take-off (**C**) and near the bifurcation carina (**D**). Lumen cross-sections appear smooth, except where internal support material was not sanded (bottom cross-section of **D**)

**Fig. 5:**
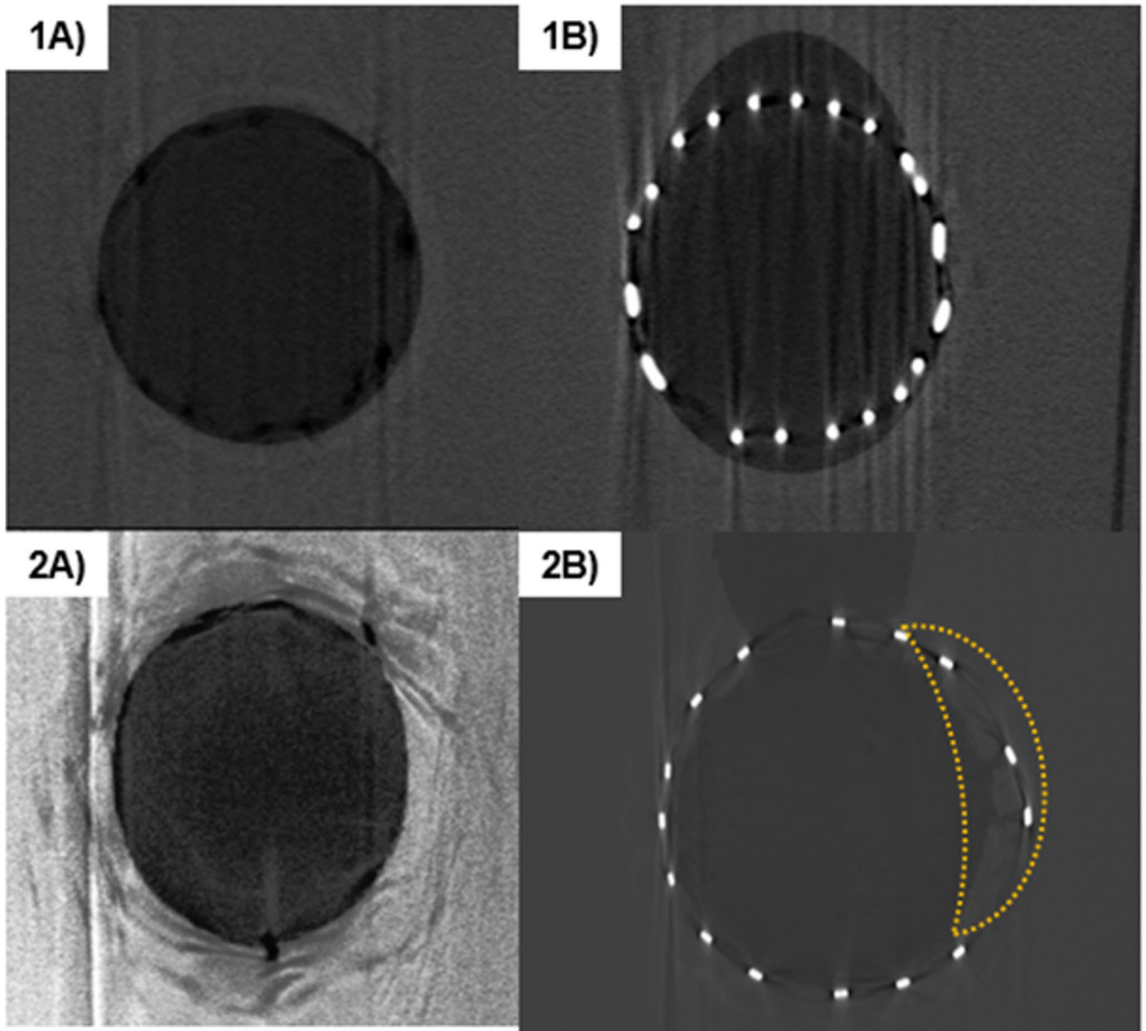
MicroCT scans of dual-layer (**1**) and single-layer phantoms (**2**). **1A** and **2A** show cross sections of the proximal vessel; the single-layer phantom was compressed into an oval shape while the dual-layer phantom maintains a circular profile. **1B** and **2B** show the phantom-stent interaction. In **2B**, the stent has torn into the single-layer phantom’s wall and the wall (outlined in yellow), with the stent (bright white) protruding into the vessel wall (light gray). **2A** and **2B** were contrast-enhanced in Image

The inner vessels held their shape and were not deformed by the weight or heat of the gel during casting. The three idealized bifurcations had a prototype cardiovascular stent placed with a prototype balloon-catheter and were microCT scanned. The resulting image stacks were manipulated using ImageJ and custom Matlab scripts to create a fly-through of the vessels, shown in Fig. 4. Figure 5 shows the microCT scans of the two-layer model compared to the single-layer model previously developed. The cross-sections of the vessel remains substantially more circular in the dual-layer version.

The vessel portion “proximal” to the stent in the models held their circular shape, such that they were used as a fiducial marker for image orientation. Image stack orientation was much more difficult with the single-layer gel phantoms, which did not hold their circular shape as well and had higher surface roughness. Because of this, the single-layer phantom’s image stacks were oriented by hand. In the dual-layer vessels, there was sufficient contrast between the stent, inner wall, and gel to measure the features of the vessel. Some halo artifacts from the stainless-steel stents were present, but images features were still clear. The vessels were smooth where the support material had been sanded off, though visually there was some discoloration where the supports had been attached to the interior walls. Additionally, we found that it was best to snip and sand the supports rather than tearing them from the wall, as in some instances this could remove a small divot in the wall.

The final phantoms were optically semi-translucent (limited by the vessel walls), as seen in Figs. 1 and 3. This allowed for easy visualization of devices within the phantom during testing.

### Tensile Testing Results

**Fig. 6:**
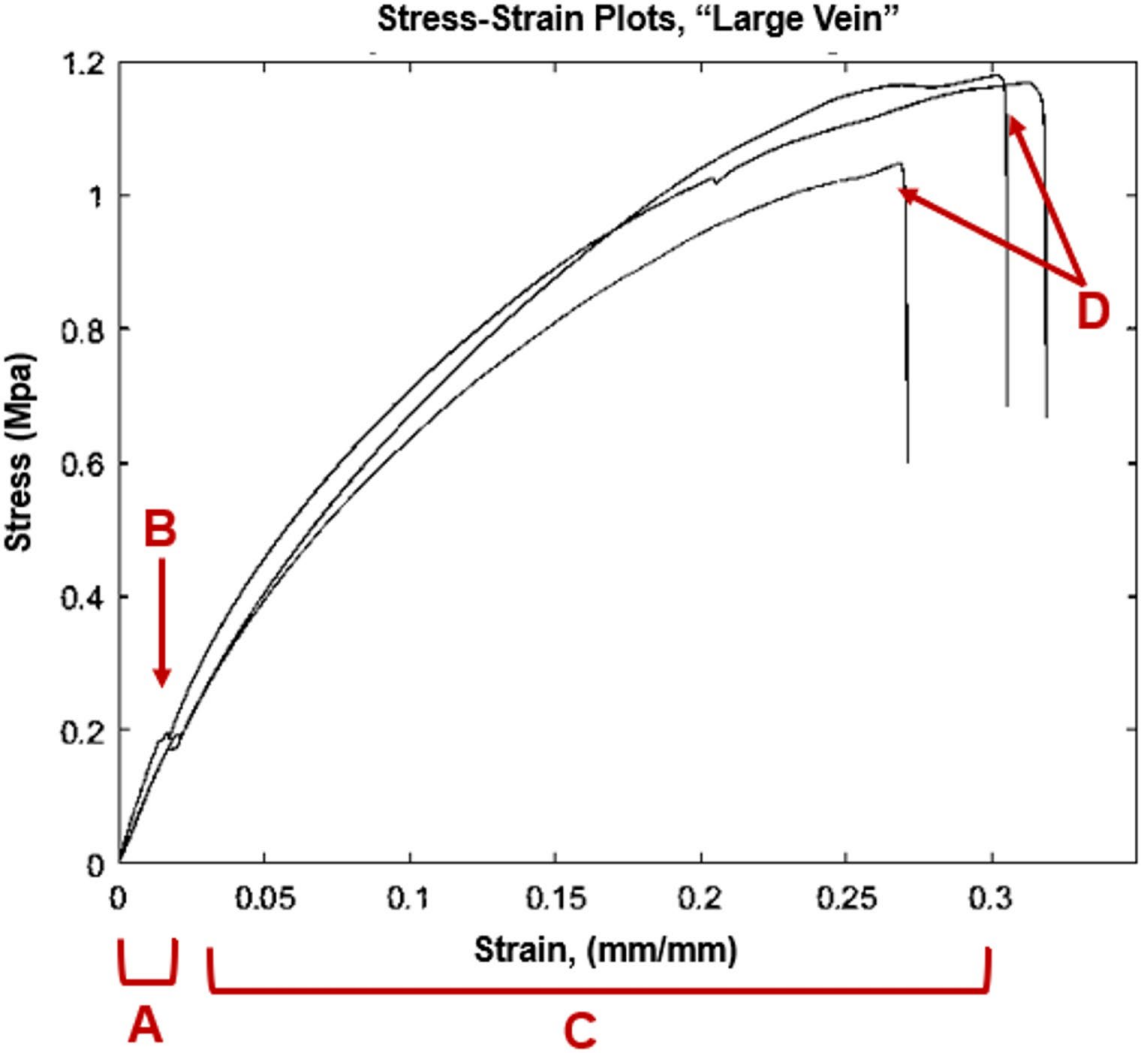
Exemplary stress/strain curves for three samples at the “Large Vein” thickness. Linear stress/strain relationship between 0–2% strain (**A**), where there is a clear yield point (**B**) leading into a region of plastic deformation (**C**). The plastic deformation is largely linear as it approaches ultimate tensile strength. There is practically no necking before break (**D**), around 25–30% strain

Thirty six specimens of elastic resin of variable size were created and tested, with 45 tests conducted total, due to slipping and retesting of 9 specimens. Engineering stress and strain were calculated for each time increment of each test, then the Elastic moduli were plotted. Sample stress-strain plots for the “large vein” thickness (1.2 mm) are shown in Fig. 6:

Observation of the resulting stress-strain curves showed a linear elastic region, a clear yield point, a large region of strain hardening, then an abrupt break. The strain hardening region tended to have a second region of linearity, so a “plastic modulus” (P) was calculated, defined as the linear portion of the stress-strain curve in the plastic deformation region. For all moduli, an $\:{r}^{2}$ value was calculated and confirmed to be $\:{r}^{2}>0.9$.

**Fig. 7:**
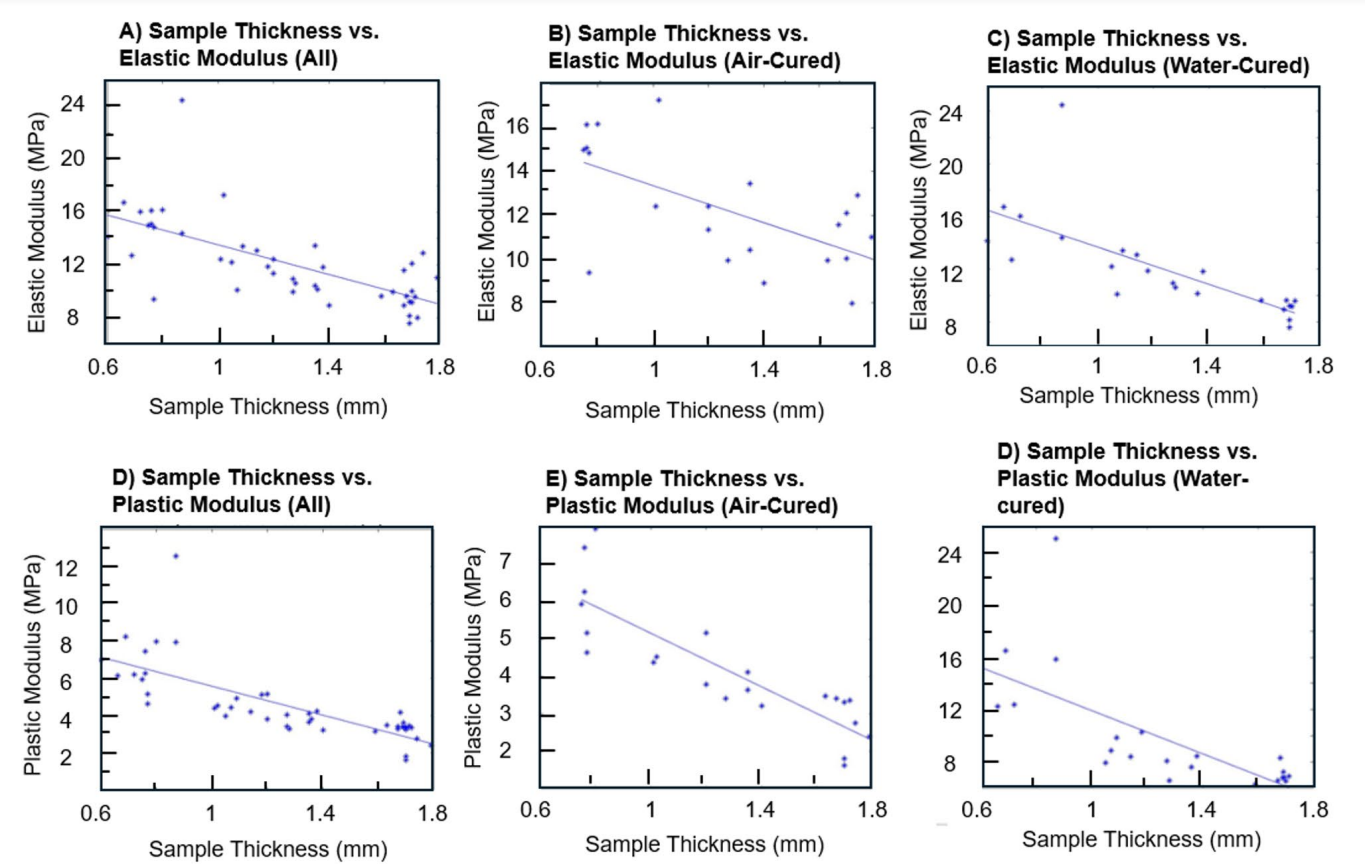
Sample thicknesses vs. elastic and plastic moduli. There was a weak negative linear relationship between sample thickness and both moduli when outliers were removed

The stress and strain at the yield point and tensile strength were also identified and recorded. Descriptive statistics are presented in Table 2, and box plots for the variables are shown in Fig. 7:

**Table 2:** Descriptive statistics for all samples, mean +/- standard deviation

Measure	Value
Yield Stress	0.21 +/- 0.074 MPa
Yield Strain	1.9 +/- 1.6%
Ultimate Tensile Strength	1.27 +/- 0.44 MPa
Elastic Modulus (E)	12 +/- 3.2 MPa
Plastic Modulus (P)	4.6 +/- 2.0 MPa
Elongation at Break	29 +/- 9%

Averaged across all samples (*n* = 45), the elastic modulus was 12 ± 3.2 (MPa). When calculated separately between water-cured and air-cured, the elastic moduli were 12 ± 3.7 and 12 ± 2.6 respectively. A two-tailed t-test was calculated between E’s, and no significant difference was observed (*p* = 0.8). Table 3 shows the average Young’s modulus for each artery thickness between water-cured and air-cured samples.

**Table 3:** Average elastic-domain young’s modulus for each blood vessel thickness and cure process

Sample Thickness (mm)	Corresponding Vessel Thickness	Air cured Young’s Modulus (MPa)	Water Cured Young’s Modulus
0.75 ± 0.09	Small Vein	15.4 ± 0.7	13.8 ± 0.9
0.76 ± 0.07	Small Artery	13.5 ± 3.6	19.1 ± 4.7
1.12 ± 0.15	Med. Vein	12.9 ± 4.2	11.8 ± 1.5
1.22 ± 0.07	Med. Artery	11.2 ± 1.2	11.7 ± 1.2
1.44 ± 0.13	Large Vein	11.3 ± 1.9	10.5 ± 1.1
1.72 ± 0.09	Large Artery	10.8 ± 1.6	8.9 ± 0.8

The average P was 4.6 ± 2.0 across all samples. For water-cured samples, the plastic modulus was 4.9 ± 2.2, and for air-cured samples *P* = 4.2 ± 1.6. A two-tailed t-test between the air-cured and water-cured samples found no significant difference (*p* = 0.19). Table 4 shows the data for P.

**Table 4:** Average plastic-domain modulus for each blood vessel thickness and cure process

Sample Thickness (mm)	Corresponding Vessel Thickness	Air Cured Plastic Modulus (MPa)	Water Cured Plastic Modulus (MPa)
0.75 ± 0.09	Small Vein	6.850 ± 1.479	9.253 ± 2.907
0.76 ± 0.07	Small Artery	5.608 ± 0.854	10.490 ± 6.194
1.12 ± 0.15	Medium Vein	4.040 ± 0.716	4.206 ± 0.240
1.22 ± 0.07	Medium Artery	4.120 ± 0.919	4.347 ± 0.842
1.44 ± 0.13	Large Vein	3.744 ± 0.329	3.730 ± 0.546
1.72 ± 0.09	Large Artery	2.669 ± 0.750	3.517 ± 0.309

To look at the effect of sample thickness on Elastic modulus, linear regression analyses were also conducted. For all samples, $\:{r}^{2}$ = 0.46. For air-cured samples only, the $\:{r}^{2}$ = 0.40. The water-cured samples saw an $\:{r}^{2}$= 0.55, but when one outlier was removed, the $\:{r}^{2}$ = 0.81. Sample thickness vs. Young’s modulus is shown in Fig. 7, between all samples, air-cured, and water-cured samples.

A linear regression analysis was calculated to estimate the effect of sample thickness on P. For all samples, $\:{r}^{2}$ = 0.57; air-cured: $\:{r}^{2}$ = 0.73 with slope = -3.6, and water-cured $\:{r}^{2}$ = 0.51. When one observed outlier was removed, $\:{r}^{2}$ for all samples was 0.71 with slope of -3.5. With the same outlier removed, the water-cured $\:{r}^{2}$ became 0.71 as well with a slope of -3.4.

Linear regressions of the effect of sample thickness on UTS and elongation at break were also conducted, with $\:{r}^{2}$= 0.55 for all samples of UTS, with a negative slope, and $\:{r}^{2}$= 0.41 for all samples of elongation with a positive slope.

## Discussion

### Vascular Phantoms

One straight vessel, three idealized bifurcations, and one patient-specific model were successfully produced using the described method. The inner lumen shape was stabilized effectively by the Aquapour, and casting the artificial gelatin over the inner lumen was also successful. The idealized bifurcation was scaled up and required internal supports; however, the vessel was large enough to remove internal supports manually, with residual bumps sanded off. The patient-specific coronary artery model has a smaller inside diameter, such that no internal supports were required for successful printing. Printing without internal supports may not be possible for all vascular phantom shapes, which is somewhat of a limitation of this method. Though printed without internal supports using a resin printer with layer height of 0.1 mm, there are still layer lines in the final phantom. Another limitation of this method over casting around a 3D printed core is to the difficulty of smoothing the layer lines, particularly on interior surfaces, while a casting core can be smoothed to a glossy finish before casting [23].

Another consideration for this method is ensuring the 3D printed lumen matches the intended design. We stiffened the vessel with the Aquapour material, and used a support for the idealized bifurcation to hold it in place (Fig. 1B). Though it wasn’t necessary for our intended uses, for more complex geometries it may be necessary to stabilize the lumen during Aquapour filling, perhaps with a multi-piece cradle. Also, a consideration for this process is that we found variations in the designed versus the actual thickness of our tensile test samples, particularly for the thinnest specimens. The manufacturer recommends printing with at least 1 mm thickness, though our printer was capable of printing to 0.75 mm consistently. Other researchers have printed down to 0.4 mm thickness, though this increased the rate of printing failures [17]. Limiting print thickness to 1 mm limits the range of producible vessel wall thicknesses to those corresponding to medium and large arteries or large veins.

### Imaging

The microCT analysis of the idealized bifurcations with imbedded stents gave clear distinction between the gel layer, the lumen layer, and the metallic stent. The relatively high density of stainless steel compared to the much lower density of the phantom materials did produce some artifacts, but overall was not an issue for imaging. Relatively simple image processing techniques using Matlab and ImageJ were used to filter and identify edge points. For patient-specific models with curvature in multiple planes, more sophisticated tools and methods may be required, though this was outside the scope of our work.

Future work will also focus on testing with multiple imaging modalities. For the interior lumens, the manufacturer description indicates that the material is translucent, and we found that following curing the materials stayed relatively clear. However, it was observed that the material yellows somewhat during the oven de-bubbling process, staying semi-transparent. The addition of the gel layer enhanced the clarity of the phantoms; this effect has been seen in other work that coated 3D-printed elastic phantoms [26]. One imaging issue for future work may be reducing the yellowing of the internal vessel, which we hypothesize can be done by reducing oven de-bubbling times and/or temperature. Other imaging techniques like ultrasound can be explored; for this, we may need to minimize the thickness of the surrounding gel block and investigate the speed of sound in the vessel material.

### Mechanical Properties

The results of our mechanical testing showed that Elastic 50 A is comparable to physiological tissue. Tables with comparative values are in Appendix I. Gel 0 has previously been characterized, and is within physiological ranges [8].

Compared to properties of human tissue, the measured UTS is comparable to the UTS of femoral arteries, the adventitia of arteries (iliac and coronary), to the normal aorta and to some varieties of aortic plaque [29, 40]. The Young’s Modulus was similar to mammary artery values, and some coronary artery values but not all [24, 40, 41]. Elongation at break was lower than desired. Together, this indicates that the interior lumen material is not unreasonable for use as an arterial substitute, though for large deformations the material may not have the required elasticity.

Human tissue is not easily described by typical engineering metrics, as each tissue type exhibits different and complex mechanical properties [28–30]. However, as most synthetic materials are linearly elastic and uniaxial testing is significantly simpler to perform than biaxial testing, it is still useful to compare Young’s modulus and UTS for arterial components.

For all mechanical properties, there was no significant difference between the water-cured and air-cured samples, indicating that the bulk of polymerization that occurs during curing is not dependent on the presence of water. The tackiness of air-cured resin may help the resin adhere to the gel during phantom assembly. Future work in this area will include testing compliance and distensibility of the gel, elastic resin, and the gel/resin combination, as the compliance of the inner lumen is likely affected by the gel surrounding it.

The thickness of the sample did have an interesting impact on the resin’s mechanical properties, with a moderate degree of the variation correlated with the sample thickness. As sample thickness increased, the Young’s moduli and UTS tended to decrease, and the elongation at break tended to increase. In other words, the thicker samples were not as stiff as the thinner samples. This may be due to the thinner samples allowing more UV penetration within the sample, resulting in a stiffer material. Other work has indicated that individual layers of resin have gradients of the amount that they cure, based on Beer-Lambert’s law of laser penetration [42]. For our thinner models, the secondary UV cure may have fully penetrated the material, eliminating the cure gradients within the material. Future work could explore the relationship between cure time and material stiffness for model tuning.

There was some variation between our mechanical test results and the results reported elsewhere. Table 5 shows the different properties reported for Elastic V1 and Elastic V2 resins from different testers.

**Table 5:** Properties of elastic resin

	Elastic V1 From [26]	Elastic V1 (Manufacturer)	Elastic V2 (Manufacturer)	Elastic V2 (Measured)
**Measurement**				
Ultimate Tensile Strength	1.35 MPa	3.23 MPa	3.4 MPa	1.27 +/- 0.44 MPa
Elastic Modulus (E)	2.41 MPa	Not reported	Not reported	12 +/- 3.2 MPa
Elongation at Break	118.62%	160%	160%	29 +/- 9%
**Test Settings**				
Print Orientation	75 degrees	Not reported	Not reported	90 degrees
Specimen Shape	dogbone	dogbone	dogbone	rectangular
Strain Rate	10 mm/min	508 mm/min	Not reported	50 mm/min

Our UTS was 1.3 MPa, which is comparable to other reported UTS (1.35 MPa), but does not agree with the manufacturer-reported 3.4 MPa UTS (Elastic V2 Material Data Sheet, Formlabs, Somerville MA). Our tests showed a Young’s modulus of 12.3 MPa, higher than other reports. We also saw a distinct linear-elastic stress-strain curve, while other reports saw a much flatter overall curve [26]. 

There are several factors that may account for these differences. First, we are using V2 of the Elastic resin, while previous studies used V1 [26]. Secondly, sample shape (rectangular strip vs. dog-bone) may contribute to variations as well., Finally, print orientation affects the mechanical properties of resin-printed parts [43]. Gradients in the degree of polymerization along the thickness of the sample are possible [42, 43]. As model complexity increases, maintaining the same print orientation for each cross-section of the printed lumen will not be possible, highlighting a need for the mechanical properties of 3-D printed materials to be assessed for various print angles and reported as a range of values.

## Conclusion

In this work, a process for describing dual-layer cardiovascular phantoms is described. We successfully created idealized and patient-specific arterial models using our described methods. However, due to limitations with 3D printing, phantoms less than 1 mm wall thickness are difficult to produce, which in turn limits phantoms to mimicking medium-to-large vessel sizes or scaled models. The final phantoms were semi-transparent and well-suited to microCT scanning. This makes these phantoms useful for a variety of biomedical applications, such as medical device testing with pressure and flow, and/or imaging studies, though additional imaging modalities have not yet been explored. The gel material has been previously validated as mimicking human tissue, and hence only the interior lumen material was tested here. The interior lumen material showed a moderate degree of flexibility but may not be soft enough for all applications.

## Data Availability

MicroCT and Instron data are available upon request to the authors.

## Appendix I: A Note on Casting Molds

Several options for casting molds were explored, and we have found several viable options. Ideal casting molds are quick to produce and customize, easy to use, impermeable, do not warp during use, and are easy to remove from the completed phantom. Originally, we used metal baking pans with holes drilled for the vessel inlets and outlets. However, we decided in this work to investigate molds that could be dissembled after casting, or were otherwise flexible to allow easier removal of the completed phantoms. This is because the dual-layer model’s interior vessel extend out from the casting pans, and we did not want to tear them during removal from the pans. Materials we tried in this work include printing molds from a high-heat resin, silicone molds purchased off-the-shelf, filament-printing a HIPS mold with low infill, and panels of polymer clay. Methods and results are now described.

The resin-printed method had two open sides for two silicone panels, which were cut from off-the-shelf silicone sheets. The two silicone panels were adhered using silicone caulk. This method worked, and was used to prepare the idealized phantoms created in this study. Two drawbacks were time of preparation, size limitations, and silicone warping. The long preparation time is due to the length of print time with resin printing (around 10 h to print, 3 additional hours for post-processing). Following printing, the molds also need sanding to smooth off layer lines which can cause the molds to stick to the completed phantoms. Resin printing also tends to be size-limited, due to platform size. The silicone panels worked, and were easy to remove from the completed phantoms, but tended to warp during the casting process. This is acceptable for larger models where the silicone panels are separated from the region of interest, but for smaller models warping may be problematic.

Silicone baking molds purchased off-the-shelf had the benefit of being cheap and quick to produce, with the only preparation being holes cut for vessel inlets and outlets. Additionally, after casting, the flexible silicone molds allowed for easy removal of the final phantom. However, the main issue with this method is the warping of the silicone, which doesn’t allow for a neat phantom block.

Filament printing with HIPS (high-impact polystyrene) was also explored. HIPS has a higher melting temperature than other 3D printable filaments, above the temperature of the gel during formation. HIPS was also quick to print (around three hours) and doesn’t require hands-on preparation. However, we found that HIPS also softens and shrinks significantly during gel heating.

Polymer clay is cheap, but requires more hands-on time to produce. Clay was rolled flat and cut into panels of the desired size (1 h), panels were baked according to manufacturer’s instructions (30 min), and assembled with silicone caulk (30 min assembly, 1 h for silicone to harden). However, this method was very effective, customizable, and not difficult to produce. Additionally, dissembling and removing the phantom required some of the least manipulation, second only to the silicone molds. The silicone caulk easily peels from the molds, and then the slabs are peeled from the phantom. We recommend the polymer clay slabs, simply because of the customizability and ease of dissembling after phantom casting. However, the high-heat resin and traditional metal casting pans are also viable options, according to operator preference.

## Appendix II: Mechanical Properties of Vascular Vessels

The mechanical properties of several tissue types and several synthetic materials have been gathered and are presented in Tables A1-A4.

**Table A1:** Ultimate tensile strength of arteries and artery-mimicking materials

Material	Tensile Strength (MPa)
*Synthetic Material*	
Elastic Resin*	1.3
PDMS βθ	3.51–7.07
Tango Plusα	0.00055–0.00103
*Arterial Components‡*	
Elastin	1–2
Collagen	120
*Normal Artery‡*	
Femoral Artery	1–2
Internal Mammary Artery	4.1 +/- 0.9 (circ) 4.3 +/- 1.8 (axial)
*Non-Atherosclerotic Aged Coronary Artery†*	
Adventitia	1.43 +/- 0.6 (circ) 1.3 +/- 0.7 (axial)
Media	0.446 +/- 0.194 (circ) 0.419 +/- 0.188 (axial)
Intima	0.394 +/- 0.223 (circ) 0.391 +/- 0.144 (axial)
*Atherosclerotic Plaques from Iliac Arteries‡*	
Surrounding Adventitia	1.0310 ± 0.306.8 (circ) 0.9518 ± 0.2090 (axial)
Non-diseased Media	0.2020 ± 0.069 (circ) 0.1888 ± 0.1109 (axial)
Non-diseased intima	0.4886 ± 0.1856 (circ) 0.9437 ± 0.2723 (axial)
Fibrous Cap	0.2548 ± 0.0798 (circ) 0.4686 ± 0.1001 (axial)
Fibrotic intima at the medial border	0.7768 ± 0.3362 (circ) 0.2775 ± 0.0984 (axial)
Diseased fibrotic media	1.0736 ± 0.2893 (circ) 0.1874 ± 0.0083 (axial)
*Human Aortic Atherosclerotic Plaques‡*	
Fibrotic Plaques	1.57 (circ) 1.64 (axial)
Calcified Plaques	1.25 (circ) 0.90 (axial)
Lipid Plaques	0.76 (circ) 0.51 (axial)
Normal Aorta	1.45 (circ) 1.35 (axial)

*Average values from mechanical testing; ‡ [40]; † [28]; § [31]; β [12]; θ [25]; α [24]

**Table A2:** Elongation at break of arteries and artery-mimicking materials

Material	Elongation at Break
*Synthetic Materials*	
Elastic Resin*	29%
Tango Plusα	170–220%
*Arterial Components‡*	
Elastin	100–150%
Collagen	13%
*Normal Artery‡*	
Femoral Artery	63–76%
Internal Mammary Artery	134 +/- 28% (circ) 59 +/- 15% (axial)

*Average values from mechanical testing; ‡ [40]; α [24]

**Table A3:** Young’s modulus for arteries and artery-mimicking materials

Material	Young’s Modulus (MPa)
*Synthetic Materials*	
Elastic Resin*	12.3
Gel 0**	0.57 (compression)
PDMS αβθ	1.32–2.97
Solaris β	0.14–0.17
PVA-c κδλ	0.03–0.31
Tango Plus α	0.34
*Arterial Components‡*	
Elastin	0.038 ± 0.004
Collagen	1000
*Normal Artery*	
Femoral Artery*‡*	9–12
Internal Mammary Artery*‡*	8.0 ± 3.0 (circ) 16.8 ± 7.1 (axial)
Right Coronary Artery αη	1.06–3.3
Coronary Arteryα	6–32

*Average values from mechanical testing; **Gel 0 material data sheet, Humimic medical, Greenville SC; ‡ [40]; α [24]; β [12]; θ [25]; λ [5]; κ [2]; δ [7] η [41]

**Table A4:** Yield stress for arteries and artery-mimicking materials

Material	Yield Stress (MPa)
*Synthetic Materials*	
Elastic Resin*	0.21 ± 0.074
*Human Cerebral Artery §*	
Cerebral Artery	1.06 ± 0.13
Aneurysm Fundi	0.5 ± 0.26
Aneurysm Neck	1.21 ± 0.49

*Average values from mechanical testing; § [31]
